# Determinants of maternal morbidity during pregnancy in urban Bangladesh

**DOI:** 10.1371/journal.pone.0268487

**Published:** 2023-02-24

**Authors:** Zakir Hossain, Nilima Afroz, Sabina Sharmin, Sayema Sharmin, Enamul Kabir

**Affiliations:** 1 Department of Statistics, University of Dhaka, Dhaka, Bangladesh; 2 Road Transport and Highways Division, Ministry of Road Transport and Bridges, Dhaka, Bangladesh; 3 School of Mathematics, Physics and Computing, University of Southern Queensland, Toowoomba, Australia; Bangladesh Agricultural University, BANGLADESH

## Abstract

**Background:**

Maternal morbidities especially life-threatening pregnancy complications are major health concerns in developing countries. The main aim is to investigate the prevalence of maternal morbidity during pregnancy and its determinants among women from urban areas of Bangladesh.

**Methods:**

The secondary data were used and extracted from the latest Bangladesh Urban Health Survey (BUHS) 2013. Several statistical models: Poisson, negative binomial (NB) and mixed Poisson were adapted and compared to explore the best model for investigating potential determinants of maternal morbidity. Pearson chi-square statistic was used for the detection of overdispersion in the data.

Results

Overall 13.5% of the urban women in Bangladesh suffered from at least two pregnancy complications. The study detected the overdispersion existing in the maternal morbidity count data and found the NB regression as the best choice for analyzing the data because of its smallest Akaike information criterion. Administrative division (Rangpur: *p* = 0.003, incidence rate ratio, IRR = 1.34, 95% confidence interval, CI: 1.11 to 1.63; Sylhet: *p* = 0.006, incidence rate ratio, IRR = 1.42, 95% CI: 1.11 to 1.82), unwanted pregnancy (p<0.001, IRR = 1.25, 95% CI: 1.11 to 1.40), place of delivery (*p*<0.001, IRR = 1.68, 95% CI: 1.53 to 1.86) and wealth index (Poor: *p*<0.001, IRR = 1.34, 95% CI: 1.19 to 1.50; Middle: *p* = 0.003, IRR = 1.21, 95% CI: 1.08 to 1.36) were found to be statistically significant determinants for maternal morbidity during pregnancy among the urban women in Bangladesh.

**Conclusions:**

Urban women in Bangladesh with an unwanted pregnancy, from the poor/middle-income group; and living in Rangpur and Sylhet divisional cities have a higher risk of maternal morbidity during pregnancy. Study findings may help the government and relevant authorities to take necessary steps for reducing maternal morbidity and mortality due to pregnancy-related complications.

## Introduction

Reproductive health care is a highly focused issue in the process development of a country. Millions of women, in developing countries like Bangladesh, experience life-threating and other health related complications during pregnancy, delivery and post-partum periods in every year. Although, pregnancy related complications leading to maternal and child mortality are common among women in Bangladesh, attention have not been drawn by the researchers and other relevant authorities to address this issue.

Like most developing countries, a rapid urbanization problem in Bangladesh is growing in recent decades as a part of the universal trend. In 2015, about 65.7% of the total population of Bangladesh was living in the rural areas, whereas, it is predicted that by 2039 the majority of the population will be living in the urban areas [1].

Approximately 5.7 million of the total population of Bangladesh (3.8%) are living in the urban slums with very limited health facilities [2]. Although, health care services are available in most urban regions of Bangladesh, the underprivileged residents living in the slums of urban areas have lesser access to important health care services compared to their wealthier counterparts. The community health centre facility was freely available only to 7.3% of the slum dwellers in Bangladesh [3]. Also, the poorest and illiterate women living in urban slums were less likely to take adequate maternal health care services from the medically skilled professionals [4].

Every year, about 9.5 million women suffer from pregnancy and delivery related complications worldwide and the number of maternal deaths is estimated to be more than 300,000 [5, 6]. In 2015, the maternal mortality rate (MMR) of Bangladesh was 176 while the targeted MMR for the millennium development goals (MDGs) was set to reduce it to 143 per 100,000 live births. This rate was substantially higher in Bangladesh compared to other countries [7]. In the 2030 sustainable development goal (SDG 3.1) the global MMR is fixed at less than 70 per 100,000 live births [8]. Thus, investigating the reasons of maternal mortality in Bangladesh is very important for further reduction in MMR targeting in SDG 3.1.

Approximately three quarters of maternal mortality in the world occurred due to pregnancy and delivery related complications [9]. About 810 women died every day in 2017 from avoidable causes of pregnancy and childbirth related complications and approximately 94% of these maternal deaths happened in low and lower middle-income countries [8]. A study on maternal mortality in Bangladesh in 2007 reported that women education and poverty were two significant factors of maternal deaths [10]. Another recent study found that early maternal age (<18 years), unwanted pregnancy, migration status and NGO (non-government organization) membership were important risk factors for pregnancy and delivery related complications among the urban women in Bangladesh [11].

Hemorrhage/severe bleeding, fits/convulsion, odema, excessive vomiting, and cough/high fever were considered as life-threatening and high risk pregnancy and delivery complications [12–15]. Thus, it is very important to investigate the associated factors of these complications in order to reduce maternal and child mortality and hence to give safe births to achieve the SDGs. Unfortunately, a limited number of studies has been conducted addressing directly the maternal morbidity/complications during pregnancy in the urban areas of Bangladesh.

The main objective of this paper is to find out the potential determinants of maternal morbidity during pregnancy among the urban women in Bangladesh using the latest Bangladesh Urban Health Survey (BUHS) 2013 data. The number of pregnancy complications was considered as the count response variable and therefore, over-dispersion (extra-variation) nature of the data has also been explored and taken into account in the current study to avoid misleading inferences and to ensure valid interpretation of the results which was entirely overlooked in the previous studies.

## Materials and methods

### Data and sampling design

We have used the secondary data on maternal morbidity during pregnancy, extracted from the latest Bangladesh Urban Health Survey (BUHS) 2013. The survey was conducted at the household level using a three-stage stratified sampling design during 23 July to 12 December, 2013 and the details are available at https://dataverse.unc.edu/. The data were collected from 1718 clusters in major urban areas, comprising 450 in City-corporation slums, 900 in City-corporation non-slums, and 368 in other urban areas of Bangladesh. In total, after excluding missing cases, records of 6001 women were used in this study who had their last birth in three years preceding the BUHS 2013. Statistical packages: SPSS (Statistical Package for the Social Sciences, Version 20, Armonk, New York, United States: IBM Corp.) and R were used for the data analysis.

### Variables included in the study

We have considered different types of pregnancy morbidities or complications, namely, haemorrhage/severe bleeding, fits/convulsion, odema, prolonged labour, high fever, leaking membrane or fluid, mal presentation, retained placenta, high blood pressure and severe headache experienced by the urban women during their last pregnancy of child birth [11, 15]. The count response of pregnancy complications is our variable of interest in this study.

To investigate the potential risk factors of maternal morbidity during pregnancy, several socio-economic and demographic variables such as; age, administrative division or place of residence, religion, education, number of ever-born children, multiple last birth, wanted pregnancy, at least 8 ANC (antenatal visits) [16, 17], place of delivery, delivery by MTP (medically trained professional), sex of last child, migration status, wealth index, NGO (non-government organization) membership and media exposure are considered as explanatory variables. The ‘wealth index’ was determined on the basis of economic status of women in the context of Bangladesh. The women came from rural areas or other cities were considered as migrant. The variables ‘media exposure’ and ‘NGO membership’ were derived by combining the associated covariates available in the survey as these were not found directly from the survey data. Women who read a newspaper or magazine or listen to radio or watch television were considered as exposed to media. Those women who were allied with any of the organizations: Grameen Bank, Bangladesh Rural Advancement Committee (BRAC), Bangladesh Rural Development Board (BRDB), Association of Social Advancement (ASA), and Proshika were considered as NGO members.

### Overdispersion

Overdispersion is a very common scenario in modelling count data. It occurs in the case of greater variability i.e. when the variance of responses in a Poisson regression model is higher than the mean. Overdispersion should be taken into account for analyzing pregnancy complications count data of women used in this study to avoid misleading inferences. Overdispersion can be detected by using the value of the Pearson chi-square (χ^2^) statistic divided by the associated degrees of freedom (df) [18]. This value is called the *dispersion* and is used as 1, >1 and <1 for equidispersed, overdispersed, and under-dispersed models, respectively. The Pearson χ^2^ statistic can be written as

$$\chi^{2}=\sum_{i=1}^{n}\frac{{\left(y_{i}-\mu_{i}\right)}^{2}}{V(\mu_{i})},$$

where *y*_*i*_ and μ_*i*_ denote observed and expected counts, respectively; *V* is known as the variance function which is equal toμ_*i*_ for Poisson and μ_*i*_+*γ*μ_*i*_^2^ for the negative binomial regression models with the dispersion parameter *γ* [18].

### Models for count data

We considered several count regression models for analyzing the maternal morbidity data of women during their pregnancy in urban Bangladesh. More precisely, Poisson regression (PR), negative binomial regression (NBR) and mixed Poisson regression (MPR) models were adapted in the context of generalized liner models (GLM) and generalized liner mixed models (GLMM) framework. The PR is the initial step for modelling count data. Let *Y*_*i*_; (*i* = 1,…,*n*) be the count response for *i*^th^ individual with mean *E*(*Y*_*i*_) = μ_*i*_, then the PR model using the *log* link-function can be written in the GLM framework [19, 20] as

$$Log(\mu_{i})=\eta_{i}=X_{i}^{T}\beta ,$$

Where *X*_*i*_ denotes a *p*×1 column vector of covariates, *β* is a1×*p* vector of regression parameters and *η*_*i*_ is called the linear predictor. The PR model is restrictive because of its equidispersion assumption of the mean and variance of count responses. However, count data are often overdispersed (extra variation) or underdispersed (less variation) compared to the PR model.

The negative binomial regression (NBR) is an alternative and widely used to model over-dispersed count data. The random effect *U* is considered to model the unobserved variability that exists in the data. It is usually assumed that *U* follows the gamma distribution mainly for computational simplicity with shape parameter *δ* and scale parameter *δ*^−1^. The variance of *Y*_*i*_ in the NBR model can be written as ${Var(Y_{i})=\mu }_{i}+\gamma {\mu_{i}}^{2}=\mu_{i}(1+\gamma \mu_{i})$ with *γ* >0 [18, 21]. The overdispersion i.e. the extra quantity of μ_*i*_ is 1+*γ*μ_*i*_, a multiplicative factor, which depends on μ_*i*_. It shows that the variance is larger than the mean and therefore, the overdispersion in the data is accounted for in the NBR model in contrast to the PR through the dispersion parameter *γ* = *δ*^−1^. When *γ* = 0, then *Var*(*Y*_*i*_)=μ_*i*_ and hence *E*(*Y*_*i*_)=*Var*(*Y*_*i*_)=μ_*i*_, which is the condition of equidispersed Poisson distribution.

The MPR is also a further improvement of modelling correlated or clustered count data, which is an extension of the PR where random effects are introduced in the linear predictor [22]. Let *Y*_*jk*_ be the *k*^th^ individual (*k*= 1,…,*n*_*j*_) under j^th^ cluster (*j* = 1,…,*m*) then similar to the PR, the MPR can be written in the context of GLMM as

$$Log(\mu_{jk})=Log(E(Y_{jk}|u_{j}))=\eta_{jk}=X_{jk}^{T}\beta +u_{j},$$

where μ_*jk*_ = *E*(*Y*_*jk*_|*u*_*j*_) is the conditional expectation and *η*_*jk*_ is the liner predictor [23, 24]. As before *Log* is the link function and *u*_*j*_ is the *j*^th^ cluster effect (*j* = 1,2,…,1718) represents the random intercept in the MPR model. Estimates of the model parameters are obtained by assuming normally distributed and uncorrelated random effects. The likelihood-based model selection criterion, Akaike information criterion (AIC), is used to select the best model with minimum AIC value and it can be defined as

$$AIC=-2l(\hat{\theta ;}y)+2p,$$

where $l(\hat{\theta ;}y)$ is the log-likelihood, $\hat{\theta }$ is the vector of estimated regression parameters and *p* is the number of model parameters of interest [25]. For the convenience of interpretation of estimated model parameters, the incidence rate ratio (IRR) associated with the individual covariate *X*_*i*_; *i* = 1,…,*p* is widely used and can be written as ${IRR}_{i}=e^{\hat{\beta_{i}}}$, where $\hat{\beta_{i}}$ is the *i*^th^ estimated regression coefficient [26].

### Ethics statement

The authors did not employ any person to collect information for this article. Bangladesh Urban Health Survey (BUHS) 2013, approved by ICF Macro Institutional Review Board and the National Research Ethics Committee of the Bangladesh Medical Research Council was used for this study. A written consent about the survey was taken from the participants by the survey authority before conducting the interview. All identification of the respondents was dis-identified before publishing data. The secondary data set used in this study is freely available online at https://dataverse.unc.edu/.

## Results

Table 1 shows that among all urban women included in this study, 3.3% (197 out of 6001) and 2.7% (161 out of 6001) experienced life-threating pregnancy complications, hemorrhage/severe bleeding and fits/convulsion, respectively. It can also be seen that 15.3% (917 out of 6001), 7.6% (455 out of 6001) and 1.2% (73 out of 6001) of Bangladeshi urban women faced high-risk pregnancy complications: odema, prolonged labor and high fever.

**Table 1:** Frequency and percentage (%) distributions of pregnancy complications among the urban women (n = 6001) in Bangladesh.

Pregnancy complications	%
Hemorrhage Severe bleeding	3.3
Fits/Convulsion	2.7
Odema	15.3
Prolonged labor	7.6
High fever	1.2
Leaking membrane	6.7
Mal presentation	1.1
Retained placenta	1.2
High blood pressure	5.5
Severe headache	12.4

In addition, we observe that 6.7% (401 out of 6001) urban women faced the complexity of leaking membrane, 1.1% (67 out of 6001) mal-presentation, 1.2% (69 out of 6001) retained placenta, 5.5% (331 out of 6001) high blood pressure and 12.4% (747 out of 6001) severe headache or blurred vision. The number of pregnancy complications/morbidities with its mean and variance were computed and summarized in Table 2.

**Table 2:** Distribution of the number of pregnancy complications among the urban women in Bangladesh.

Distribution	Number of pregnancy morbidities/complications						Total
	0	1	2	3	4	5+	
Frequency	3851	1339	515	185	72	39	6001
%	64.2	22.3	8.6	3.1	1.2	0.6	100

Mean = 0.57 and variance = 0.90 (i.e. extra variation)

Table 2 reveals that 22.3% (1339 out of 6001) of the urban women in Bangladesh experienced one and 13.5% at least two complications during their pregnancy. Bivariate analysis was carried out considering several socio-economic, demographic and biological variables associated with pregnancy complications using the ANOVA (analysis of variance) F-test (Table 3). Administrative division (*p*<0.001), education (*p* = 0.032), wanted pregnancy (*p<*0.001), place of delivery (*p<*0.001), wealth index (*p<*0.001) and NGO membership (*p<*0.001) of women were found to be statistically significant at 5% level with their mean number of pregnancy complications.

**Table 3:** Bivariate analysis of the socio-economic and demographic variables along with 95% confidence interval (CI) for mean number of pregnancy complications along with *p*-values of the ANOVA *F*-tests.

Variable	Mean morbidity	95% CI	*p*-value
Age (years)			0.505
≤20	0.59	(0.53, 0.64)	
>20	0.57	(0.54, 0.59)	
Administrative division			**<0.001**
Dhaka	0.57	(0.53, 0.60	
Barisal	0.72	(0.56, 0.88)	
Chittagong	0.49	(0.44, 0.53)	
Khulna	0.62	(0.52, 0.72)	
Rajshahi	0.56	(0.48, 0.65)	
Rangpur	0.80	(0.68, 0.92)	
Sylhet	0.75	(0.57, 0.94)	
Religion			0.520
Non-Muslim	0.54	(0.46, 0.63)	
Muslim	0.57	(0.55, 0.60)	
Education			**0.032**
Primary	0.61	(0.56, 0.65)	
Secondary	0.57	(0.54, 0.60)	
Higher	0.51	(0.47, 0.56)	
Ever born children			0.979
1 or 2	0.57	(0.54, 0.60)	
3 or higher	0.57	(0.51, 0.63)	
Multiple last birth			0.438
No	0.70	(0.30, 1.10)	
Yes	0.57	(0.54, 0.59)	
Wanted pregnancy			**<0.001**
No	0.71	(0.64, 0.79)	
Yes	0.55	(0.52, 0.57)	
At least 8 ANC visits			0.160
No	0.57	(0.54, 0.59)	
Yes	0.65	(0.53, 0.76)	
Media exposure			0.297
No	0.62	(0.52, 0.71)	
Yes	0.57	(0.54, 0.59)	
Delivery by MTP			**<0.001**
No	0.53	(0.50, 0.56)	
Yes	0.63	(0.59, 0.66)	
Sex of last child			0.655
Girl	0.56	(0.53, 0.60)	
Boy	0.57	(0.54, 0.61)	
Migration status			0.925
Migrant	0.57	(0.54, 0.60)	
Non-migrant	0.57	(0.53, 0.60)	
Wealth index			**<0.001**
Poor	0.62	(0.58, 0.66)	
Middle	0.58	(0.53, 0.63)	
Rich	0.50	(0.46, 0.53)	
NGO membership			**<0.001**
No	0.55	(0.52, 0.57)	
Yes	0.68	(0.61, 0.75)	
Place of delivery			**<0.001**
Health facility	0.65	(0.62, 0.68)	
Home	0.43	(0.39, 0.46)	

ANC: Antenatal care, MTP: Medically trained professional, NGO: non-government organization

To find out the factors associated with pregnancy complications among the urban women in Bangladesh, we selected the best model (Table 4) and conducted multivariable analysis (Table 5), using selected variables that were found to be statistically significant at 5% level in bivariate analysis (Table 3). From Table 2, it can be seen that mean and variance of the count responses (number of pregnancy complications/morbidities) were 0.57 and 0.90 respectively, that is extra variation exists in the data. It was investigated whether the over-dispersion was present in pregnancy complications count data used in this study (Table 4). The PR model was first fitted to detect overdispersion by computing the *Dispersion* value using the Pearson chi-square statistic. Table 4 reveals that the *Dispersion* value for the PR model was 1.53 (greater than 1), which clearly depicts the presence of overdispersion and hence, the PR model was found to be overdispersed.

**Table 4:** Detection of overdispersion and model selection for analyzing pregnancy complications count response data from multivariable Poisson regression (PR), negative binomial regression (NBR) and mixed Poison regression (MPR) models.

Multivariable models	Detection of overdispersion and model selection	
	Dispersion	AIC
PR	1.53	12665.84
**NBR**	**1.01**	**12181.60**
MPR	1.02	12359.63

**Table 5:** The results of the final multivariable negative binomial regression model to identify maternal morbidity determinants during pregnancy in urban Bangladesh.

Variables	Estimate	p-value	IRR	95% CI for IRR
Administrative Division				
Dhaka*				
Barisal	0.18	0.172	1.19	(0.93, 1.54)
Chittagong	-0.09	0.117	0.92	(0.82, 1.02)
Khulna	-0.01	0.978	1.00	(0.89, 1.17)
Rajshahi	-0.06	0.491	0.94	(0.80, 1.12)
Rangpur	0.30	**0.003**	1.34	(1.11, 1.63)
Sylhet	0.35	**0.006**	1.42	(1.11, 1.82)
Wanted pregnancy				
Yes*				
No	0.22	**<0.001**	1.25	(1.11, 1.40)
Wealth index				
Rich*				
Middle	0.19	**0.003**	1.21	(1.08, 1.36)
Poor	0.29	**<0.001**	1.34	(1.19, 1.50)
Place of delivery				
Home*				
Health facility	0.52	**<0.001**	1.68	(1.53, 1.86)

Note:

* indicates reference category

We then fitted both the NBR and MPR models for further improvement of modelling the overdispersed pregnancy complications count data used in this study. From *Dispersion* and AIC values (Table 4), it was observed that the overdispersion was well captured and modelled by the NBR compared to the PR and MPR models because of its smallest AIC (12181.60) and *Dispersion* (1.01) values. We also computed the variance inflation factor (VIF) to detect the presence of multicollinearity and found that all VIF values are less than 10 (1<VIF<2). This indicates the absence of significant multicollinearity in the data [27]. The results obtained from fitting the best selected model (NBR) were summarized in Table 5.

From Table 5, it is observed that the urban women from Rangpur (*p =* 0.003, IRR = 1.34, 95% CI: 1.11 to 1.63) and Sylhet (*p =* 0.006, IRR = 1.42, 95% CI: 1.11 to 1.82) divisional regions were more likely to develop pregnancy complications than women from Dhaka city. Wanted pregnancy (*p*<0.001, IRR = 1.25, 95% CI: 1.11 to 1.40) was strongly associated with their average number of pregnancy complications/morbidities. As expected, the women who did not desire the index pregnancy were more likely to experience complications during pregnancy compared to the women who desired it. The women from poor (*p*<0.001, IRR = 1.34, 95% CI: 1.19 to 1.50) and middle (*p =* 0.003, IRR = 1.21, 95% CI: 1.08 to 1.36) income groups were more likely to suffer from pregnancy complications/morbidities than the women who belong to the rich-income group.

Place of delivery (*p*<0.001, IRR = 1.68, 95% CI: 1.53 to 1.86) was significantly associated with pregnancy complications of the women from urban areas of Bangladesh.

## Discussion and conclusion

Like other developing countries, a significant number of the urban women of Bangladesh experience major high-risk and life-threatening complications/morbidities during their pregnancy and delivery periods. In this study, our main aim is to investigate the prevalence and determinants of maternal morbidities/complications of urban women during pregnancy in Bangladesh.

This study findings reveal that 3.3% and 2.7% of the urban women faced life-threatening pregnancy and delivery-related complications/morbidities, hemorrhage/severe bleeding, and fits/convulsion, respectively. Overall, 13.5% of the women experienced at least two complications during their last childbirth. Overdispersion was detected in the count responses of pregnancy complications and therefore, the data were analyzed by fitting the best model, negative binomial regression, because of its minimum AIC value. Whether desired the index pregnancy, place of residence or administrative division, place of delivery, and wealth index of the urban women were found to be significant risk factors for maternal morbidity during pregnancy in Bangladesh. Similar findings were also observed in previous studies [10, 11] except for covariates: age and children ever born.

The risk of experiencing maternal morbidities or pregnancy complications was higher among the pregnant women who belong to the poor/middle-income group. This scenario is common among Bangladeshi women because of not taking sufficient antenatal care for safe pregnancy and childbirth due to their wealth constraints. The women who did not want the index pregnancy were at high risk of facing complications during pregnancy due to the lack of proper preparation and care about the pregnancy. The higher risk of maternal morbidity was evident among women living in Rangpur or Sylhet regional cities because of limited modern health facilities compared to the capital city Dhaka. It is surprising that women who give birth at health centers are more likely to have complications compared to their counterparts giving birth at home. This may happen because those urban women in Bangladesh usually visit health centres when they already suffered from pregnancy-related complications.

We used the data on maternal morbidity collected in 2013 by the Urban Health Survey as no further survey was conducted yet in Bangladesh, which is the limitation of the study. This study recommends that governmental and nongovernmental organizations, policymakers, and other relevant authorities should focus on the awareness of antenatal health care for women to reduce pregnancy and delivery-related complications, and hence maternal and child deaths.
